# Human EWS-FLI protein levels and neomorphic functions show a complex, function-specific dose–response relationship in *Drosophila*

**DOI:** 10.1098/rsob.240043

**Published:** 2024-07-17

**Authors:** Serena Mahnoor, Cristina Molnar, Diego Velázquez, Jose Reina, Salud Llamazares, Jan Peter Heinen, Jaume Mora, Cayetano Gonzalez

**Affiliations:** ^1^ Institute for Research in Biomedicine (IRB Barcelona), The Barcelona Institute of Science and Technology, Barcelona, Spain; ^2^ Developmental Tumor Biology Laboratory, Institut de Recerca Sant Joan de Déu, Esplugues de Llobregat, Barcelona, Spain; ^3^ Pediatric Cancer Center Barcelona (PCCB), Hospital Sant Joan de Déu, Esplugues de Llobregat, Barcelona, Spain; ^4^ Institucio Catalana de Recerca i Estudis Avançats (ICREA), Barcelona, Spain

**Keywords:** Ewing sarcoma, Ewing study model, EWS-FLI, GGAA microsatellite

## Abstract

Ewing sarcoma (EwS) is a cancer that arises in the bones and soft tissues, typically driven by the Ewing’s sarcoma breakpoint region 1-Friend leukemia virus integration 1 (EWS-FLI) oncogene. Implementation of genetically modified animal models of EwS has proved difficult largely owing to EWS-FLI’s high toxicity. The EWS-FLI_1FS_ frameshift variant that circumvents toxicity but is still able to perform key oncogenic functions provided the first study model in *Drosophila*. However, the quest for *Drosophila* lines expressing full-length, unmodified EWS-FLI remained open. Here, we show that EWS-FLI_1FS_’s lower toxicity is owed to reduced protein levels caused by its frameshifted C-terminal peptide, and report new strategies through which we have generated *Drosophila* lines that express full-length, unmodified EWS-FLI. Using these lines, we have found that the upregulation of transcription from GGAA-microsatellites (GGAAμSats) presents a positive linear correlation within a wide range of EWS-FLI protein concentrations. In contrast, rather counterintuitively, GGAAμSats-independent transcriptomic dysregulation presents relatively minor differences across the same range, suggesting that GGAAμSat-dependent and -independent transcriptional upregulation present different kinetics of response with regards to changing EWS-FLI protein concentration. Our results underpin the functional relevance of varying EWS-FLI expression levels and provide experimental tools to investigate, in *Drosophila*, the effect of the EWS-FLI ‘high’ and ‘low’ states that have been reported and are suspected to be important for EwS in humans.

## 1. Introduction

Ewing sarcoma (EwS) is an aggressive tumour, typically arising from bone and soft tissues, which is reported to be the second most common bone malignancy in children, adolescents and young adults [1–3].

EwS is a paradigm for solid tumour development caused by a single genetic lesion, a fusion between the transactivation domain of any member of the FET (FUS, EWSR1 and TAF15) family of RNA-binding proteins and the DNA-binding domain of one of several E26 transformation specific (ETS) transcription factors (reviewed in [2]). In more than 80% of patients, the actual proteins involved are EWS RNA-binding protein 1 (EWSR1) and Friend leukaemia integration 1 (FLI1) and the resulting fusion is the EWS-FLI oncogene [2,4,5]. About 60% of these fusions have breakpoints at EWSR1 exon 7 and FLI1 exon 6 (i.e. type 1) henceforth referred to as EWS-FLI_1_ [6–8].

EWS-FLI is a pleiotropic oncoprotein with diverse neomorphic functions that include the remodelling of transcriptionally silent micro-satellites made of GGAA repeats (GGAAμSats) into active neo enhancers, out competition of ETS transcription factors and forcing aberrant alternative splicing [9–14]. Through these functions, EWS-FLI establishes the oncogenic regulatory program that subverts normal cell physiology and drives EwS development (reviewed in [2]).

The overwhelming toxicity of EWS-FLI has hampered the development of genetically tractable models of EwS and successful attempts to express it in vertebrates are still rare [15,16].

In *Drosophila melanogaster*, EWS-FLI toxicity can be avoided by a frameshift mutant (EWS-FLI_1FS_) that replaces the 69 amino acid C-terminal tail of EWS-FLI with a new 69 amino acid sequence (henceforth referred to as the FStail) [17]. Remarkably, upon expression in *Drosophila*, EWS-FLI_1FS_ interacts with the homologues of EWS-FLI’s human protein partners including subunits of RNA pol II, the BAP/BAF chromatin remodelling complex and others; and causes a massive dysregulation of the transcriptome that affects a fraction of the *Drosophila* homologues of human EwS signature genes [17]. Indeed, such a massive transcriptional dysregulation in *Drosophila* must be largely GGAAμSat-independent because, unlike the human genome that contains thousands of GGAAμSats of optimal length (i.e. 18× to 26×(GGAA) [18,19]), the *Drosophila* reference genome (r6.40) contains less than one hundred of which the longest is 9×(GGAA). However, as in EwS cells, human EWS-FLI can also convert silent GGAAμSats into neoenhancers in *Drosophila* as shown with transgenic lines carrying 20 GGAA repeats upstream a heat-shock minimal promoter (HSP) followed by the yellow fluorescent protein (YFP)-coding sequence, (*20×(GGAA)μSat>YFP* [17]). Moreover, human EWS-FLI is also capable of outcompeting ETS transcription factors and causing aberrant alternative splicing in *Drosophila* [17].

Altogether, these results showed that genetically engineered *Drosophila* strains expressing EWS-FLI_1FS_ may serve as genetically tractable study models to investigate EWS-FLI function. However, the question of how the FStail reduces toxicity remained open and so was the quest for a *Drosophila* line capable of expressing the unmodified full-length EWS-FLI protein.

Here, we show that EWS-FLI_1FS_’s reduced toxicity is owed to decreased protein concentration brought about by the FStail. Building on this observation, we have generated new lines carrying transgenes designed to downregulate transcription or translation, hence, avoiding the need for FStail tagging. Using these lines, it is possible to achieve a wide range of full-length, unmodified EWS-FLI_1_ protein levels that result in a complex phenotypic series. From these series, we have found that activities of EWS-FLI_1_ like assembly of neo-enhancers at GGAAμSats and GGAAμSat-independent transcriptomic dysregulation respond differently to varying amounts of EWS-FLI.

Altogether, our results reveal a complex, phenotype-specific, dose–response relationship between the amount of EWS-FLI_1_ protein and its neomorphic functions, which in turn implies fundamental differences in the corresponding underlying mechanisms.

## 2. Results

### 2.1. The C-terminal peptide of the EWS-FLI_1FS_ variant results in decreased protein levels

The frameshift variant EWS-FLI_1FS_ was discovered as a serendipitous mutant that circumvented embryonic lethality, which was presumably caused by leaky expression from the Upstream Activating Sequence (UAS) of *UAS>EWS-FLI* (i.e. expression in the absence of any Gal4 driver). Functional assays showed that rather than to the loss of the 69 amino acid C-terminal peptide of EWS-FLI_1_, the lower toxicity of EWS-FLI_1FS_ was owing to a neomorphic property of the new FStail [17]. We did not know the nature of such a neomorphic property, but a simple hypothesis was that it could be owing to reduced protein expression and/or stability.

**Figure 1.**
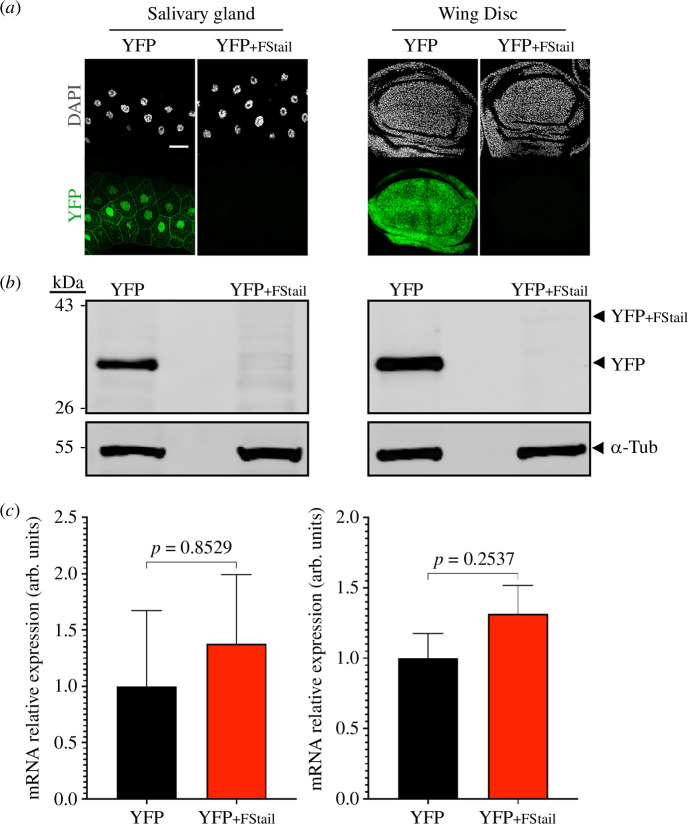
The FStail reduces protein levels without affecting transcription. YFP protein levels observed by fluorescence (*a*) and western blot (*b*), and YFP mRNA quantified by RT-qPCR (*c*) in salivary glands (left) and wing imaginal discs (right) from *nub>Gal4/UAS>YFP*; (YFP) and *nub>Gal4/UAS>YFP_+FStail_* (YFP_+FStail_) larvae. Scale bar = 50 µm.

To test this hypothesis, we quantified the effect of FStail tagging on YFP. To this end, we generated transgenic animals carrying *UAS>YFP* and *UAS>YFP_+FStail_*, a fusion of the FStail to the C-terminal end of YFP. By driving *UAS>YFP* and *UAS>YFP_+FStail_* expression from the *nub>Gal4* driver, we found that both YFP_+FStail_ green fluorescence intensity, as measured by confocal microscopy, as well as YFP_+FStail_ protein levels, as quantified by western blot, are extremely reduced compared with those of unmodified YFP in salivary glands and wing imaginal discs (figure 1*a,b*).

Because the YFP and YFP_+FStail_ transgenes were inserted at the same genomic site (attp40 [20]), by φC31 integrase-mediated transgenesis [21] and were both expressed by *nub>Gal4*, transcription levels are expected to be comparable. That is unless the FStail-coding sequence affects transcription, which could indeed explain the differences observed at the protein level. To test this hypothesis, we quantified YFP and YFP_+FStail_ transcripts by reverse transcription-quantitative polymerase chain reaction (RT-qPCR) in salivary glands and wing imaginal discs. We found no significant differences in the level of mRNA from each variant in either tissue (figure 1*c*). Altogether, these results show that the FStail has no significant effect on transcription but affects protein expression and/or stability such that it results in a strong reduction in the concentration of a protein carrying this C-terminal tag. These results substantiate the hypothesis that EWS-FLI_1FS_’s reduced toxicity is owing to a significant reduction in protein concentration caused by the FStail.

**Figure 2.**
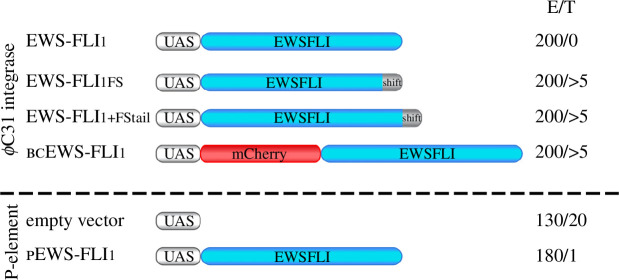
*Drosophila* transgenic lines expressing full-length human EWS-FLI type 1 protein (EWS-FLI_1_). The number of injected embryos ‘E’ and transgenic animals recovered ‘T’ in one round of injection. Transgenesis experiments with full-length EWS-FLI_1_, the published frameshift variant (UAS>EWS-FLI_1FS_), full-length EWS-FLI_1_ fused with the C-terminal peptide created by the EWS-FLI_1FS_ frameshift (UAS>EWS-FLI_1+FStail_), and a bicistronic construct carrying mCherry followed by full-length EWS-FLI (UAS>bcEWS-FLI_1_) were carried out using φC31 integrase. The P-element expressing full-length EWS-FLI_1_ (UAS>pEWS-FLI_1_) was obtained by P-element-mediated transgenesis.

To further substantiate this conclusion, and to get a direct estimate of the effect of the FStail in EWS-FLI_1_, we generated the EWS-FLI_1+FStail_ transgene, an FStail-tagged version of full-length EWS-FLI_1_. We found that in stark contrast with unmodified EWS-FLI_1_, EWS-FLI_1+FStail_ transgenes were obtained at the standard rate of more than five per 200 injected embryos, thus confirming that the sole addition of the FStail can circumvent the toxicity issue of unmodified EWS-FLI_1_ (figure 2).

### 2.2. Generating *Drosophila* strains carrying unmodified, full-length EWS-FLI transgenes

Having shown that EWS-FLI_1FS_’s reduced toxicity is owing to a significant reduction in protein concentration caused by the FStail, we decided to test if the same effect could be achieved with transgenes designed to downregulate translation or transcription, so that full-length, untagged EWS-FLI_1_ could be expressed. We used two approaches to this aim.

The first approach was to downregulate translation by placing an EWS-FLI_1_ coding sequence lacking its own internal ribosome entry site as the secondary open reading frame (ORF) in a bicistronic transcript encoding mCherry as primary ORF. Under these conditions, translation of the secondary ORF is so severely compromised that it has been estimated to be essentially null in the absence of Gal4, hence avoiding potential toxicity issues caused by leaky transcription during transgenesis [22–25].

The second approach was based on P-element-mediated transgenesis [26,27]. Unlike precise targeting to predetermined genomic sites by φC31 integrase [21], P-element transgenes insert nearly randomly in the genome and are, therefore, subjected to unpredictable position effects that may significantly affect transcription. We reasoned that this inherent drawback could be to our advantage because it may be used to select for insertions in genomic regions in which leaky *UAS>EWS-FLI_1_* expression is downregulated below toxic levels.

We found that both approaches work well to generate *Drosophila* lines carrying unmodified, full-length EWS-FLI_1_ transgenes. φC31 integrase-mediated transgenic lines carrying the bicistronic construct bcEWS-FLI_1_ were obtained at the standard rate of more than five per 200 injected embryos, thus showing that this strategy abrogates EWS-FLI_1_ embryo toxicity. P-element-mediated transgenesis was much less efficient, producing a single pEWS-FLI transgenic line out of 180 injected embryos (<1%), which is one order of magnitude below the normal rate of P-element-mediated transgenesis (>10%) (figure 2). Such a low rate is not surprising, however, since only the small fraction of P-element insertions subjected to position effects strong enough to abrogate toxic, leaky EWS-FLI_1_ expression can be expected to generate viable transgenic animals. DNA sequencing of the new transgenic lines obtained from both approaches confirmed the presence of non-mutated, full-length EWS-FLI_1_, as intended, thus validating the two strategies.

### 2.3. The collection of full-length EWS-FLI transgenes causes a range of developmental phenotypes

**Figure 3.**
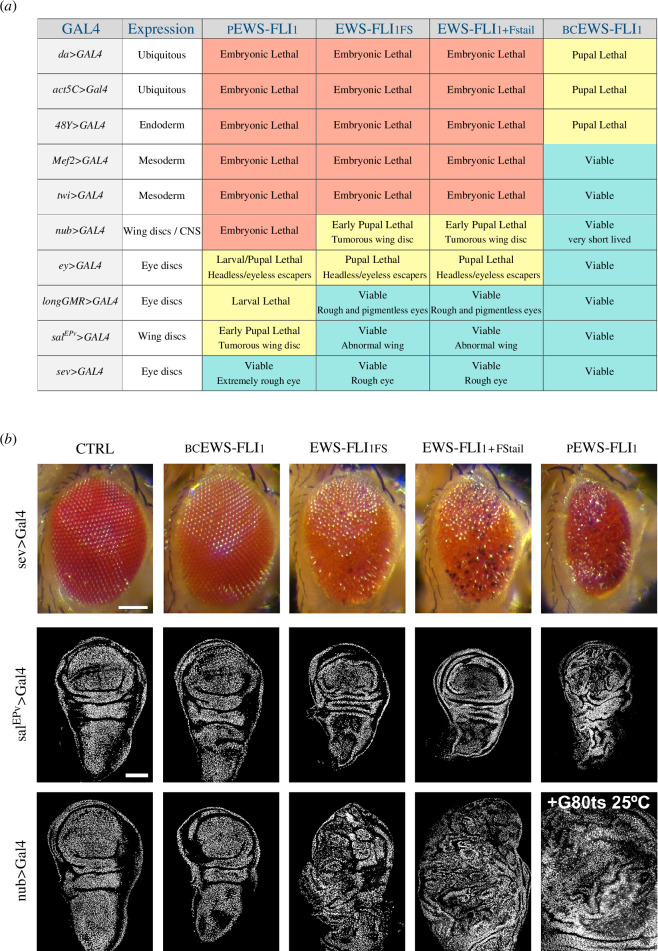
*Drosophila* transgenic lines expressing full-length human EWS-FLI_1_ generate a wide range of developmental phenotypes. (*a*) Summary of developmental phenotypes observed in each EWS-FLI_1_ transgenic line, colour coded with red for embryonic lethal, yellow for larval or pupal lethal and cyan for adult viable. (*b*) Adult eye and third instar wing imaginal discs from individuals expressing the indicated versions of EWS-FLI_1_ in ommatidia (*sev-Gal4*), central region of the wing pouch (*sal^EPv^>Gal4*) or wing pouch and hinge region (*nub>Gal4*). The *nub>Gal4/UAS>EWS-FLI_1FS_* and *nub>Gal4/UAS>EWS-FLI_1+FStail_* wing disc tumours shown are extreme examples to demonstrate the extent of transformation that can be induced by these transgenes; milder phenotypes have been observed. Most *nub>Gal4/UAS>pEWS-FLI_1_; P[tubP>GAL80^ts^]/+* individuals reared at 25°C die at early stages of development; only two third instar larval escapers that we have observed presented large wing disc tumours (+Gal80 ts 25°C). Scale bars: 0.1 mm or 100 µm for adult eyes and wing discs, respectively.

As a first step to characterize the new transgenic lines, we investigated their effect on development upon expression from a variety of Gal4 drivers (figure 3*a*). We found that as far as the lethality phase, wing disc tumours and cuticular phenotypes in adult flies are concerned, the new variants cause a wide range of expressivity levels.

Top of the range is pEWS-FLI_1_ that is lethal when expressed from any of the selected Gal4 drivers, including *longGMR>Gal4* and *sal^EPv^>Gal4* with which all of the other transgenes are viable. At the bottom of the range is the bcEWS-FLI_1_ variant that yields pupal lethality with *da>Gal4*, *Act5C>Gal4* and *48Y>Gal4*, and adult viability with *Mef2>Gal4* and *twi>Gal4*, while the same Gal4 drivers result in embryonic lethality with all other EWS-FLI_1_ versions. Moreover, surviving adults carrying bcEWS-FLI_1_ and *Mef2>Gal4* or *twi>Gal4* present no noticeable cuticular or lifespan phenotypes. EWS-FLI_1FS_ and EWS-FLI_1+FStail_ have an intermediate effect, stronger than bcEWS-FLI_1_ and weaker than pEWS-FLI_1_ (figure 3*a*). EWS-FLI_1FS_ and EWS-FLI_1+FStail_ are largely indistinguishable except for the effect on eye roughness and size when driven from *sev>Gal4*, which is stronger in the case of EWS-FLI_1+FStail_ (figure 3*b*, row 1).

To ameliorate the strong lethal effect of pEWS-FLI_1_, we also generated individuals carrying this construct together with a second transgene, *P[tubP>GAL80^ts^]*, which expresses the temperature-sensitive allele of the yeast Gal80 protein from the *alphatubulin84B* promoter. Gal80 is an inhibitor of the transcriptional activation domain of Gal4. When expressed in *Drosophila*, Gal80^ts^ is fully functional at 17°C, but it is inactive at 29°C. By raising individuals carrying the pEWS-FLI_1_ and *P[tubP>GAL80^ts^]* transgenes at 25°C we hoped to achieve a partial inactivation of Gal80 function that would in turn partially inhibit Gal4, hence lowering EWS-FLI_1_ expression from *UAS>pEWS-FLI*_*1*_. We refer to this combination as pEWS-FLI_1_+Gal80@25.

The stronger effect of pEWS-FLI_1_ compared with that of EWS-FLI_1FS_ and EWS-FLI_1+FStail_ is clearly reflected in their tumorigenic potential in wing imaginal discs. As published [17], EWS-FLI_1FS_ causes wing imaginal disc tumours when driven by *nub>Gal4*, but not by *sal^EPv^>Gal4*, which is expressed at a later stage in development and over a much smaller area in the wing disc. The same is true for EWS-FLI_1+FStail_. In contrast, *sal^EPv^>Gal4*-driven expression is sufficient for pEWS-FLI_1_ to cause wing imaginal disc tumours, and *nub>Gal4*-driven expression of pEWS-FLI_1_+Gal80@25 yields even larger tumours in the rare escapers (figure 3*b*, rows 2 and 3).

### 2.4. Upregulation of transcription from (GGAA)μSat and tissue toxicity present a positive linear correlation with a wide range of EWS-FLI protein concentrations

To further characterize the new full-length EWS-FLI_1_ lines, we quantified the efficiency of each transgene at activating transcription from GGAAμSats, which is one of the hallmarks of the EWS-FLI_1_ oncogene and is known to be critical for EwS tumorigenesis [9,18,28,29]. The *20×(GGAA)μSat>YFP* transgene was used to track GGAAμSat activation [17]. We chose to carry out these studies on third instar larval salivary glands because they are among the tissues that are more resilient to the detrimental effects of EWS-FLI_1_ [17]. EWS-FLI_1_ transgenes were expressed from *sal^EPv^>Gal4* that, like other Gal4 drivers derived from the P{GawB} vector (Flybase FBtp0000352), contains a cryptic larval salivary-gland-specific enhancer element [30].

**Figure 4.**
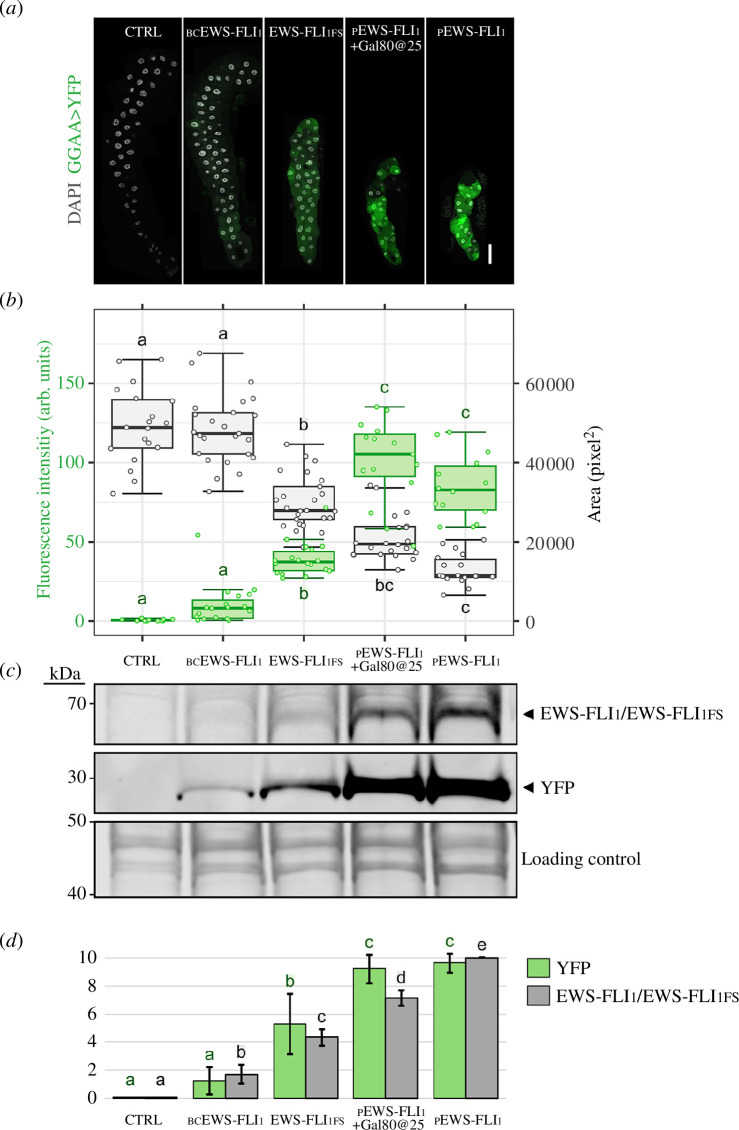
The expressivity of EWS-FLI_1_-induced phenotypes correlates with protein concentration. (*a*) Representative salivary glands showing YFP expression from *20×(GGAA)>YFP* (green) and co-stained with DAPI (grey). Scale bar: 100 µm. (*b*) Quantification of YFP fluorescence (green) and salivary gland size (grey) (arb. units). Letters indicate statistically significant differences between group means (Kruskal−Wallis and Dunn’s test, *p*<0.05). (*c*) Western blot showing protein levels of EWS-FLI_1_ and YFP; means ± s.d. of relative protein band intensities from three replicates are represented in (*d*). Letters indicate statistically significant differences between group means (ANOVA and Tukey’s HSD, *p*<0.05). Experimental conditions include: control (*sal^EPv^>Gal4/+*), bcEWS-FLI_1_ (*sal^EPv^>Gal4/UAS>bcEWS-FLI_1_; 20×(GGAA)>YFP/+*), EWS-FLI_1FS_ (*sal^EPv^>Gal4/UAS>EWS-FLI_1FS_; 20×(GGAA)>YFP/+*), pEWS-FLI_1_ (*sal^EPv^>Gal4/UAS>EWS-FLI_1_;20×(GGAA)>YFP/+*) and pEWS-FLI_1_+Gal80@25 (*sal^EPv^>Gal4/UAS>EWS-FLI_1_; P[tubP>GAL80^ts^]/20×(GGAA)>YFP*).

Using confocal microscopy, we found that the extent of YFP upregulation induced by the collection of EWS-FLI_1_ transgenes from the *20×(GGAA)μSat>YFP* construct spans over a wide range (figure 4*a,b*). At one extreme, pEWS-FLI_1_ and pEWS-FLI_1_+Gal80@25 cause the strongest effect (with means ± s.d. of 85 ± 20 arb. units, and 101 ± 26 arb. units, respectively). At the other extreme, bcEWS-FLI_1_ induces very low, but measurable levels (10 ± 12 arb. units). EWS-FLI_1FS_ results in intermediate levels of GGAAμSat transcriptional activation (39 ± 11 arb. units): 386% (*p* = 4.4 × 10^−2^) and 46% (*p* = 3.6 × 10^−2^) of that caused by bcEWS-FLI_1_ and pEWS-FLI_1_, respectively. These results show that the extent of YFP activation induced by each transgene can be ordered into a range as follows: control<bcEWS-FLI_1_<<EWS-FLI_1FS_ << pEWS-FLI_1_+Gal80@25 ≈ pEWS-FLI_1_.

We next quantified salivary gland sizes, measured as the area of the largest confocal z-section, as a proxy for tissue toxicity (figure 4*a,b*). We found that salivary gland size is indistinguishable between wild-type and *bcEWS-FLI_1_* larvae (49k ± 10k px^2^ and 48k ± 9k px^2^, respectively; *p =* 0.9), but is severely compromised by pEWS-FLI_1_, alone or in combination with *P[tubP>GAL80^ts^]* (13k ± 4k px^2^ and 22k ± 7 k px^2^, respectively). Differences between pEWS-FLI_1_ and pEWS-FLI_1_+Gal80@25 are not significant, although pEWS-FLI_1_+Gal80@25 yields a notable number of salivary glands with an intermediary size between that of pEWS-FLI_1_ and EWS-FLI_1FS_. As before, EWS-FLI_1FS_ results in intermediate levels of salivary gland sizes (30k ± 7k px^2^) that are approximately 62% (*p* = 5.6 × 10^−4^) and 230% (*p* = 3.7 × 10^−4^) the size of those from bcEWS-FLI_1_ and pEWS-FLI_1_ individuals, respectively. The absence of detrimental effects of bcEWS-FLI_1_ on salivary glands is interesting considering that it is sufficient for driving transcription from the *20×(GGAA)μSat>YFP*. These results show that, altogether, the transgenes induce toxicity as follows: control ≈ bcEWS-FLI_1_<<EWS-FLI_1FS_ < pEWS-FLI_1_+Gal80@25≤pEWS-FLI_1_.

We then carried out western blot to quantify the level EWS-FLI_1_ protein expressed through each of the EWS-FLI_1_ transgenes (figure 4*c*). As compared with EWS-FLI_1FS_, the levels of EWS-FLI_1_ protein are about 1.5 and 2.0 times higher in pEWS-FLI_1_+Gal80@25 and pEWS-FLI_1_, respectively, thereby validating the approach of raising *P[tubP>GAL80^ts^]*-carrying individuals at 25°C to tune down EWS-FLI_1_ expression from *UAS>pEWS-FLI_1_*. In contrast, EWS-FLI_1_ is hardly detectable in bcEWS-FLI_1_ individuals. Thus, we found that EWS-FLI levels linearly increase along the following range: control<bcEWS-FLI_1_<<EWS-FLI_1FS_ << pEWS-FLI_1_+Gal80@25<pEWS FLI.

Using the same western blots, we re-assessed the levels of YFP expression from the *20×(GGAA)μSat>YFP* brought about by each of the EWS-FLI_1_ transgenes and found them to be largely consistent with those observed by confocal microscopy. This includes the fact that despite approximately 40% higher EWS-FLI_1_ levels in pEWS-FLI_1_ compared with pEWS-FLI_1_+Gal80@25 individuals, YFP expression levels are similar, hence suggesting that, as far as GGAAμSat transcriptional activation is concerned, EWS-FLI_1_ levels are near saturation in pEWS-FLI_1_+Gal80@25 individuals.

Altogether, these results demonstrate a tight correlation of EWS-FLI protein levels to both their detrimental effect on salivary gland development and their effect on upregulating transcription from (GGAA)μSats.

### 2.5. GGAAμSat-independent transcriptome dysregulation is not linearly correlated with EWS-FLI protein levels

In spite of its frame-shifted C-terminal tail, EWS-FLI_1FS_ expression recapitulates in *Drosophila* key oncogenic functions of EWS-FLI_1_ like transcriptional dysregulation of hundreds of genes including a fraction of the orthologues of known EWS-FLI’s targets in human cells [17]. The question, however, remains open as to whether unmodified full-length EWS-FLI_1_ protein could bring about a transcriptomic signature that is closer to that of EwS tumours than the EWS-FLI_1FS_’s signature.

To address this question, we used Affymetrix microarrays to perform a genome-wide transcriptomic analysis of salivary glands carrying the *bcEWS-FLI_1_*, and pEWS-FLI_1_+Gal80@25 transgenes that express very different levels of full-length EWS-FLI_1_ (electronic supplementary material, table S1). EWS-FLI_1FS_, salivary glands were also included as reference. For each condition, we determined the total number of dysregulated transcripts, quantified the enrichment of two published EwS signatures, and assessed the upregulation of neural genes, a feature that has been reported upon expression of EWS-FLI in human cells [31,32].

**Figure 5.**
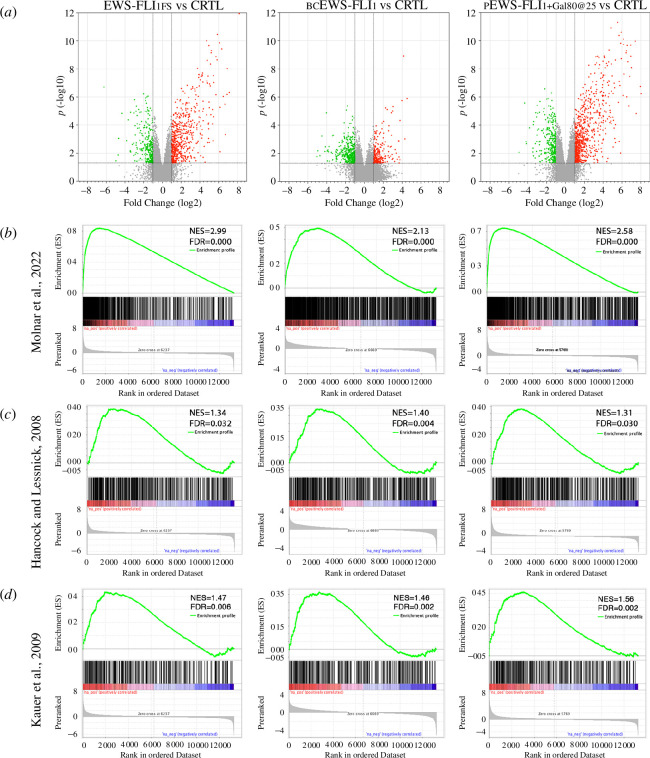
GGAAμSat-independent transcriptome dysregulation is not linearly correlated with EWS-FLI protein levels. (*a*) Volcano plots showing changes in gene expression levels in EWS-FLI_1FS_ (*sal^EPv^>Gal4/UAS>EWS-FLI_1FS_*), bcEWS-FLI_1_ (*sal^EPv^>Gal4/UAS>bcEWS-FLI_1_*), and pEWS-FLI_1_+Gal80@25 (*sal^EPv^>Gal4/UAS>EWS-FLI_1_; P[tubP>GAL80^ts^] /+*) compared withcontrol (*sal^EPv^>Gal4/+*) samples Red and green dots represent genes that are significantly (*p* < 0.05) up (FC > 2) and downregulated (FC < −2) genes, respectively. (*b–d*) Gene set enrichment analysis showing the *Drosophil*a Molnar *et al*., 2020 [17] (*b*) and the human Hancock and Lessnick, 2008 [33] (*c*) and Kauer *et al*., 2009 [34] (*d*) EwS signatures to be significantly enriched (FDR < 0.25) in the ranked datasets of EWS-FLI_1FS_, bcEWS-FLI_1_ and pEWS-FLI_1_+Gal80@25 compared with control.

We found that although the total number of genes affected in pEWS-FLI_1_+ Gal80@25 (*n* = 879) and EWS-FLI_1FS_ (*n* = 849) is larger than in bcEWS-FLI_1_ (*n* = 561), all three conditions cause a strong dysregulation of transcription (figure 5*a*) and the resulting transcriptomes are all highly significantly enriched for the published EWS-FLI_1FS_ *Drosophila* signature [17] (figure 5*b*). Moreover, with regards to upregulated genes, approximately one-third of the 404 genes of the Hancock and Lessnick signature [33] were significantly enriched at the top of all three gene set enrichment analysis (GSEA)-ranked *Drosophila* datasets: EWS-FLI_1FS_ (*n* = 115; FDR *q* = 0.032), bcEWS-FLI_1_ (*n* = 117; FDR *q* = 0.004) and pEWS-FLI_1_+Gal80@25 (*n* = 125; FDR *q* = 0.030) (figure 5*c*). Similar results were obtained with the 277 genes of the Kauer *et al*. signature [34]: EWS-FLI_1FS_ (*n* = 74; FDR *q* = 0.006), bcEWS-FLI_1_ (*n* = 82; FDR *q* = 0.002) and pEWS-FLI_1_+ Gal80@25 (*n* = 101; FDR *q* = 0.02). However, differences between bcEWS-FLI_1_ and the other conditions became apparent with regard to the upregulation of genes associated with neural development, a trait of EWS-FLI_1_ activity in human cells that is also observed in *Drosophila* salivary glands expressing EWS-FLI_1FS_ [17]. Gene ontology (GO) biological process ‘nervous system’ is well represented in both the pEWS-FLI_1_+Gal80@25 and the EWS-FLI_1FS_ transcriptomes (*n* = 121, *p* = 6.8 × 10^−18^; and *n* = 69, *p* = 8.2 × 10^−5^), but is not significantly enriched in the bcEWS-FLI_1_ transcriptome with only 13 genes from the same GO being upregulated.

These are remarkable results as far as bcEWS-FLI_1_ is concerned, taking into account the extremely low levels of EWS-FLI_1_ protein and the lack of toxicity caused by this construct that nonetheless is nearly as good as EWS-FLI_1FS_ at driving GGAAμSat-independent transcriptome dysregulation. Altogether, these results show that, unlike GGAAμSat-dependent transcriptional upregulation, GGAAμSat-independent transcriptome dysregulation, including ectopic expression of orthologues of human EwS signature genes, is not linearly correlated with EWS-FLI_1_ protein levels.

## 3. Discussion

EWS-FLI cytotoxicity has proven a great hindrance in the development of genetically tractable animal models [15]. In *Drosophila*, a very clear distinction can be made between toxicity caused by *UAS>EWS-FLI_1_* in the absence of Gal4 and toxicity caused by Gal4-driven expression. The former, which accounts for the extreme difficulty in generating transgenic lines, can be circumvented by tagging the EWS-FLI_1_ fusion with the FStail [17]. The latter, however, is only partially reduced by the FStail. Here, we show that the FStail works by reducing protein levels, which in turn suggests that FStail tagging may facilitate the implementation of other animal models expressing EWS-FLI and perhaps other lethal oncogenic fusion proteins.

Building on this observation, we have generated new lines that express full-length, unmodified EWS-FLI_1_ from transgenes that were engineered to reduce protein expression to a greater or lesser extent, hence circumventing the need for FStail tagging.

Using the new EWS-FLI_1_ lines, it is possible to generate a phenotypic series, from which we have learned that the neomorphic effects brought about by EWS-FLI_1_ that we have studied can be categorized in three classes on the basis of how they correlate with EWS-FLI_1_ protein levels. The first includes the assembly of neo-enhancers at GGAAμSats that presents a positive linear correlation over a wide range of EWS-FLI_1_ protein levels, from the lowest, (bcEWS-FLI_1_) up to the highest (pEWS-FLI_1_+Gal80@25 and pEWS-FLI_1_). The second includes massive GGAAμSat-independent transcriptional dysregulation including, notably, a significant fraction of orthologues of human EwS signature’ genes. Rather counterintuitively, despite the order of magnitude lower level of expressed EWS-FLI_1_, the bcEWS-FLI_1_ transgene is able to significantly dysregulate hundreds of genes albeit with lower fold changes. Finally, transcriptional upregulation of neural genes, a landmark of EWS-FLI activity in human cells, is only marginal in bcEWS-FLI_1_ compared with EWS-FLI_1FS_ and pEWS-FLI+Gal80@25. These results reveal a complex, phenotype-specific correlation between EWS-FLI_1_ levels and the expressivity of its phenotypes, that, in turn, implies fundamental differences in the mechanisms that drive these phenotypes. In particular, our results suggest that GGAAμSat-dependent and GGAAμSat-independent transcriptional upregulation present different kinetics with regard to EWS-FLI protein concentration, opening a new perspective on EWS-FLI-dependent dysregulation of transcription.

Despite a low mutational burden characteristic of paediatric tumours, EwS displays high levels of intratumoral heterogeneity largely mediated by alterations to the epigenetic state. Several independent findings have suggested that differences in EWS-FLI levels are a determinant of different cellular phenotypes that may have direct implications for EwS progression [35–43]. Altogether, our results highlight the functional relevance of EWS-FLI expression levels and provide experimental tools to further investigate in *Drosophila* the molecular pathways affected by the EWS-FLI ‘high’ and ‘low’ states observed in human tumours.

## 4. Material and methods

### 4.1. Fly stocks

The following fly strains were used in this study: *sal^EPv^>Gal4* [44], *tub>Gal80*^ts^ (BDSC#: 7018), *UAS>EWS-FLI_1FS_*, *UAS>EWS-FLI_1+FStail_*, *UAS>uORF-EWS-FLI_1_ (UAS>bcEWS-FLI_1_*)*, P[UAS>EWS-FLI_1_]* (***p****EWS-FLI_1_)* and *20×(GGAA)>YFP*. All Gal4 lines in figure 3*a* are described in Flybase. The wild-type strain used was (*w^1118^*). All crosses, including controls, were maintained at 25°C.

### 4.2. Genotypes and crossing schemes

For the microarray expression profiling experiment, females *w; sal^EPv^>Gal4* were crossed to males *w^1118^*, to males *w; UAS>EWS-FLI_1FS_/CyO* and to males *w; UAS>bcEWS-FLI_1_/CyO* to obtain *sal^EPv^>Gal4/+* (CTRL), *sal^EPv^>Gal4/UAS>EWS-FLI_1FS_* and *sal^EPv^>Gal4/UAS>bcEWS-FLI_1_* samples. To obtain FL samples, females *w; sal^EPv^>Gal4; P[tubP>GAL80^ts^]* were crossed to males *w^1118^; P[UAS>EWS-FLI_1_]/CyO, S, Tb*.

For quantification of 20×(GGAA)>YFP fluorescence, EWS-FLI_1_ protein levels and salivary gland size, females *w; sal^EPv^>Gal4; 20×(GGAA)>YFP/TM6B* were crossed to males *yw*, *UAS>EWS-FLI_1FS_ /CyO, to males; UAS>bcEWS-FLI_1_,* and to males *w; P[UAS>EWS-FLI_1_]/CyO,S,Tb*, to obtain *sal^EPv^>Gal4/UAS>EWS-FLI_1FS_; 20×(GGAA)µSat>YFP/+, sal^EPv^>Gal4/UAS>bcEWS-FLI_1_; 20×(GGAA)>YFP/+,* and *sal^EPv^>Gal4/P[UAS>EWS-FLI_1_]; 20×(GGAA)>YFP/+*, respectively. For pEWS-FLI_1_+Gal80@25, females *w; sal^EPv^>Gal4; P[tubP>GAL80^ts^]* were crossed to males *P[UAS>EWS-FLI_1_]/CyO; 20×(GGAA)>YFP/TM6B* to obtain *sal^EPv^>Gal4/P[UAS>EWS-FLI_1_]; 20×(GGAA)>YFP/P[tubP>GAL80^ts^]*. All these crosses were maintained at 25°C. Wandering third instar larvae from each cross were dissected, fixed and mounted to visualize the 20×(GGAA)>YFP expression in the salivary glands under the confocal microscope. All the experiments were performed blind. All individuals analysed were between 4 and 6 days old.

### 4.3. Immunohistochemistry and microscopy

Immunostaining of salivary glands and wing discs was performed as follows: salivary glands and wing discs were dissected in phosphate-buffered saline (PBS), fixed for 30 min in 4% formaldehyde with 0.3% TritonX-100, washed three times in PBS-0.3% Triton X-100 (PBST) for 10 min per wash. DNA was stained with DAPI. Salivary glands and wing discs were mounted in Vectashield (molecular probes). Immunostaining images were acquired with a Leica TCS SP8 scan unit coupled to a microscope and managed by Leica Application Suite X (LAS X) software. The objective used was HC PL APO CS2 40×/1.30 OIL and images were acquired at zoom 1. Fluorophores 405 and 488 were excited with lasers diode and argon, respectively. Image processing was carried out with Fiji. All shown immunofluorescence images correspond to a single Z.

### 4.4. Quantification and statistical analysis

*Quantification of YFP fluorescence levels* was carried out using ImageJ to calculate the mean grey values of each region of interest (ROI) in a focal plane per salivary gland acquired with an SP8 Leica confocal image microscope.

*Quantification of salivary gland size* was performed in Inkscape 1.2.2 using Bezier curves to manually outline individual salivary glands from the distal end till the meeting point of the individual ducts at the proximal end and measure the area of the resulting shape through the visualized path extension.

Both salivary gland size and YFP intensity were represented in boxplots, *p*-values for salivary gland size and GGAAμSat activation were calculated in R version 4.2.3. by Dunn’s test with Holm’s method for *p*-value adjustment after rejection of Kruskal–Wallis one-way ANOVA and compact letter display (CLD) was used to represent *p*-values on boxplots. Values of *p* for western blot band intensities were calculated by Tukey’s HSD after the rejection of one-way ANOVA.

### 4.5. Cloning and transgenesis

The pUASt P-element EWS-FLI_1_ construct was made by cloning the *EcoRI-XhoI* fragment from pUC-GW-Kan EWS-FLI_1_ TI that contains the full EWS-FLI_1_ ORF in the original pUASt vector [45]. The pUAST-EWS-FLI_1+FStail_ construct was made by introducing a *BbsI* site into the pUC-GW-Kan EWS-FLI_1_ TI. This was achieved by replacing the *BsrGI-XhoI* fragment with a gBlock (IDT) containing the *BbsI* site at the terminus of the ORF. Subsequently, another gBlock (IDT) with the FS sequence was inserted in-frame with EWS-FLI_1_ into the *BbsI* and *XhoI* sites. The resulting fragment was subcloned into the *EcoRI* and *XhoI* sites of *pUASt-attB* (DGRC Stock 1419 [46]). The pUASt-uORF-EWS-FLI_1_ construct was made by PCR amplification of the mCherry ORF from the *pLT3-Dam* vector [24] and the EWS-FLI_1_ ORF from pUC-GW-Kan EWS-FLI_1_ TI. Subsequently, both fragments were fused and cloned by in-fusion (Takara) into *EcoRI* and *XhoI* sites of the *pUASt-attB* vector.

All PCR amplifications were performed using KOD polymerase (Merck). Cloning was performed by using In-Fusion Snap Assembly (Takara). Primers were ordered to Sigma. DNA fragments were ordered to IDT.

Transgenic fly lines were generated by BestGene Inc. (φC31 integrase-mediated transgenesis) using the Bloomington stock 24482, and by the Institute for Research in Biomedicine (IRB) Barcelona *Drosophila* Injection Service (P-element-mediated transgenesis) using the *w^1118^* line.

All transgenic lines used for experiments were sequenced to confirm that they carry the correct transgene.

gBlock DNA fragments used in this article:

BbsI.FS.XhoI: TCAGAAGAAGACCCTACTACGAGCTACCATGCCCACCAGCAGAAGGTCAACTTCGTGCCACCACATCCGTCCTCCATGCCAGTGACGTCCAGCAGCTTCTTTGGCGCTGCTTCCCAGTATTGGACGTCCCCGACCGGCGGCATCTACCCAAACCCAAACGTCCCACGCCATCCGAACACCCACGTCCCATCCCATCTGGGCTCCTACTACTGACTCGAGTAAGCA

EWS-FLI_1_ BbSi-XhoI:

GTCCAGCATGTACAAGTACCCGTCCGACATTAGCTACATGCCGAGCTACCATGCCCACCAGCAGAAGGTCAACTTCGTGCCACCACATCCGTCCTCCATGCCAGTGACGTCCAGCAGCTTCTTTGGCGCTGCTTCCCAGTATTGGACGTCCCCGACCGGCGGCATCTACCCAAACCCAAACGTCCCACGCCATCCGAACACCCACGTCCCATCCCATCTGGGCTCCTACTACGTCTTCCTCGAGTATTAG

#### 4.5.1. Microarray processing

Dissected *Drosophila* salivary glands were collected in 45 µl of a lysis buffer containing 20 mM dithiothreitol (DTT), 10 mM Tris-HCl pH 7.4, 0.5% sodium dodecyl sulfate (SDS) and 0.5 µg µl^−1^ proteinase K, incubated at 65°C for 15 min and immediately frozen until processing. RNA extraction and cDNA generation was performed at the IRB Barcelona Functional Genomics Core Facility. Briefly, RNA was treated with DNAse I and purified using magnetic beads (RNAClean XP, Beckman Coulter). RNA was quantitated with Qubit RNA HS Assay kit (Invitrogen), and RNA integrity was assessed with the Bioanalyzer 2100 RNA Pico assay (Agilent). Twenty-five nanograms of RNA were reverse transcribed and amplified using the whole transcriptome amplification method (WTA2, Sigma Aldrich) with 17 cycles of amplification. cDNA was further purified using a spin column (PureLink Quick PCR Purification Kit, Invitrogen) and quantified using a microvolume spectrophotometer (Nanodrop ND-1000, Thermo-Fisher Scientific).

For microarray processing, 8 µg of cDNA were fragmented and labelled according to the manufacturer’s instructions (GeneChip Mapping 250K Nsp Assay Kit, Affymetrix). Array hybridization was performed using the GeneChip Hybridization, Wash and Stain Kit (Applied Biosystems). Briefly, libraries were denatured at 99°C for 2 min before incubation into the *Drosophila* Genome 2.0 arrays (Applied Biosystems). Libraries were hybridized on the arrays for 16 h at 45°C for 60 rpm at GeneChip Hybridization Oven 645 (Affymetrix/ThermoFisher Scientific). Washing and Stain steps were performed using a GeneChip Fluidics Station 450 following the *Drosophila* Genome 2.0 protocol (Affymetrix/ThermoFisher Scientific). Finally, arrays were scanned with a GeneChip Scanner GCS3000 (Affymetrix/ThermoFisher Scientific). The CEL files containing the microarray data were generated with the Command Console software (Affymetrix/ThermoFisher Scientific) and were used for probeset-based gene expression measurements using robust multichip average (RMA) normalization. Results were analyzed using the Transcriptome Analysis Console 4.0 (TAC) software. Genes with an absolute FC of >2 and an *p* < 0.05 were considered differentially expressed.

#### 4.5.2 Gene set enrichment analysis

The GSEA pre-ranked algorithm was used to compare the human EWS-FLI signatures from [33,34] to all genes in the *Drosophila* microarrays ranked by average log2FC. Genesets with an FDR *q*-value of <0.25 were accepted as a significant enrichment. *Drosophila* orthologues of these human signatures were identified using the Drosophila RNAi Screening Center Integrative Ortholog Prediction Tool (DIOPT) orthologue mapping online resource [47].

#### 4.5.3. GO analysis

Functional annotation of GO terms was performed using the online tool database and annotation, Visualization and integrated discovery (DAVID) [48]. GOTERM_BIOLOGICAL_PROCESS_4 terms with a *p* < 0.05 were accepted as a significant enrichment.

#### 4.5.4. Quantitative real-time PCR

Dissected *Drosophila* larval salivary glands were collected in 45 µl of a lysis buffer containing 20 mM DTT, 10 mM Tris-HCl pH 7.4, 0.5% SDS and 0.5 µg µl^−1^ proteinase K, incubated at 65°C for 15 min and immediately frozen until processing. RNA extraction and cDNA generation were performed at the IRB Barcelona Functional Genomics Core Facility. Briefly, RNA was treated with DNAse I and purified using magnetic beads (RNAClean XP, Beckman Coulter). RNA was quantitated with Qubit RNA HS Assay kit (Invitrogen), and integrity was assessed with the Bioanalyzer 2100 RNA Nano assay (Agilent). Twenty-five nanograms of RNA were reverse transcribed and amplified using the whole transcriptome amplification method (WTA2, Sigma Aldrich) with 17 cycles of amplification. cDNA was further purified using a spin column (PureLink Quick PCR Purification Kit, Invitrogen) and quantified using a microvolume spectrophotometer (Nanodrop ND-1000, Thermo-Fisher Scientific). cDNA yield ranged from 18 to 44 µg. PowerUp SYBR Green Master Mix (Thermo Fisher Scientific) was used for quantitative real-time PCR following manufacturer’s instructions. The real-time assays were conducted in a QuantStudio 6 Flex real-time PCR system (Thermo Fisher Scientific) using SYBR green as the detection system and ROX as the reference dye. Two different pairs of primers were designed to amplify YFP cDNA. mRNA levels were assessed from three independent RNA extractions and three technical replicates were performed on each sample. RNA levels were normalized to Ribosomal Protein L32 (*RpL32)* housekeeping gene. The primers used are shown in table 1.

**Table 1.** Primers used in this article.

name	sequence
**cloning**	
EI-Cherry-FW	AGGGAATTGGGAATTCATGGCAACTAGC
Cherry-RV	GGCCATGTTATTATGCGGTACCAGAACCTTTG
EWS-FLI-FW	TAATAACATGGCCTCCACCGATTATTCCACGTAC
EWS-FLI-XhoI-RV	TAGAGGTACCCTCGAGTCAGTAGTAGGAGCCCAG
**qPCR**	
YFP-1-FW	AAGCAGAAGAACGGCATCAA
YFP-1-RV	GGGGGTGTTCTGCTGGTAGT
YFP-2-FW	ATGGTGAGCAAGGGCGAG
YFP-2-RV	TGAACTTGTGGCCGTTTACGT
RpL32-b-FW	TGTGAATTTTCCTTGTCGCGT
RpL32-b-RV	TGGATAGAGATACATTCACGCATA
**sequencing**	
HSP-FW	GCAACTACTGAAATCTGCCAAG
EWS500-FW	ACCCGATGCAACCAGTGA
EWS1100-RV	CAATGCCATGGAAGTGG
SV40	GGCATTCCACCACTGCTCCC

### 4.6. Western blot

Dissected *Drosophila* larval salivary glands or wing discs were collected in 50 µl of cold PBS. PBS was removed after dissection and the samples were frozen at −20°C. For the detection of YFP_+FStail_ and YFP, eight pairs of salivary glands or 10 wing discs were used per sample from individuals with genotypes *nub>Gal4/UAS-YFP_+FStail_* and *nub>Gal4/UAS-YFP,* respectively. For detection of EWS-FLI_1_, salivary gland pairs were collected from individuals of the genotype *sal^EPv^>Gal4/P[UAS>EWS-FLI_1_]; 20×(GGAA)>YFP/ +, sal^EPv^>Gal4/P[UAS>EWS-FLI_1_]; 20×(GGAA)>YFP/ P[tubP>GAL80^ts^], sal^EPv^>Gal4/UAS>EWS-FLI_1FS_; 20×(GGAA)>YFP/ +, sal^EPv^>Gal4/UAS>bcEWS-FLI_1_; 20×(GGAA)µSat>YFP/+,* or *sal^EPv^>Gal4/+; 20×(GGAA)>YFP/+* and loaded protein quantity was adjusted to be equal to that from 4 *sal^EPv^>Gal4/+* salivary gland pairs.

Samples were resuspended in 30 µl of 2 × SDS NuPAGE lithium dodecyl sulfate (LDS) sample buffer with 100 mM DTT, lysed via manual grinding and sonication with Bioruptor. Boiled lysates were resolved by 4–12% sodium dodecyl-sulfate polyacrylamide gel electrophoresis (SDS-PAGE) (Bolt 4–12% Bis-Tris, 1.0 mm, REF: NW04120BOX; Invitrogen), and proteins were transferred to nitrocellulose membranes (0.2-µm pore size, Invitrogen). YFP_+FStail_ and YFP proteins were detected by incubating with anti-GFP primary antibodies overnight at 4°C (mouse anti-GFP) (1:250, REF: 11814460001; Roche, Basel, Switzerland). α-tubulin (Clone Dm1A) protein was detected by incubating with rat anti-TubDM antibody (1:500, REF: T6199; Sigma Aldrich) and was used as the loading control. EWS-FLI_1_ proteins were detected by incubating with rabbit anti-FLI primary antibody (1:200, ab133485, Abcam) and mouse anti-EWS (1:500, sc-48404, Santa Cruz Biotechnology) overnight at 4°C. Total protein was detected with REVERT Total Protein Stain Kit (LI-COR) and was used as a loading control. Immunoblots were developed using Alexa Fluor 790 and Alexa Fluor 680 secondary antibodies (1:2500, Jackson ImmunoResearch) or IRDye 800CW (1:2500, LI-COR) with the Odyssey Infrared Imaging System (LI-COR).

## Data Availability

Supporting data have been included in the electronic supplementary material [49].
